# Mindful Eating Mobile Health Apps: Review and Appraisal

**DOI:** 10.2196/12820

**Published:** 2019-08-22

**Authors:** Lynnette Nathalie Lyzwinski, Sisira Edirippulige, Liam Caffery, Matthew Bambling

**Affiliations:** 1 Centre for Online Health School of Medicine University of Queensland Wooloongabba Australia

**Keywords:** feeding behavior, mindfulness, mHealth, diet

## Abstract

**Background:**

Mindful eating is an emerging area of research for managing unhealthy eating and weight-related behaviors such as binge eating and emotional eating. Although there are numerous commercial mindful eating apps available, their quality, effectiveness, and whether they are accurately based on mindfulness-based eating awareness are unknown.

**Objective:**

This review aimed to appraise the quality of the mindful eating apps and to appraise the quality of content on mindful eating apps.

**Methods:**

A review of mindful eating apps available on Apple iTunes was undertaken from March to April 2018. Relevant apps meeting the inclusion criteria were subjectively appraised for general app quality using the Mobile App Rating Scale (MARS) guidelines and for the quality of content on mindful eating. A total of 22 apps met the inclusion criteria and were appraised.

**Results:**

Many of the reviewed apps were assessed as functional and had moderate scores in aesthetics based on the criteria in the MARS assessment. However, some received lower scores in the domains of information and engagement. The majority of the apps did not teach users how to eat mindfully using all five senses. Hence, they were scored as incomplete in accurately providing mindfulness-based eating awareness. Instead, most apps were either eating timers, hunger rating apps, or diaries. Areas of potential improvement were in comprehensiveness and diversity of media, in the quantity and quality of information, and in the inclusion of privacy and security policies. To truly teach mindful eating, the apps need to provide guided examples involving the five senses beyond simply timing eating or writing in a diary. They also need to include eating meditations to assist people with their disordered eating such as binge eating, fullness, satiety, and craving meditations that may help them with coping when experiencing difficulties. They should also have engaging and entertaining features delivered through diverse media to ensure sustained use and interest by consumers.

**Conclusions:**

Future mindful eating apps could be improved by accurate adherence to mindful eating. Further improvement could be achieved by ameliorating the domains of information, engagement, and aesthetics and having adequate privacy policies.

## Introduction

### Background

Research shows that mindful eating may assist with various eating disorders, such as binge eating on excess calories, and with weight management [1,2]. Stress is also reduced when individuals take the time to be mindful while eating by relaxing, being fully present, and not thinking about their problems [3].

Mindfulness is a form of meditation involving a heightened awareness of the present moment [4]. Mindful eating is an informal mindfulness practice and involves tuning into one’s 5 senses, including sight, taste, sounds, and smells of the food, all done while being fully present and eating slowly [4-8]. The key is to follow one’s internal bodily cues rather than external eating triggers such as stress or emotional eating and to cease eating when one feels full instead of overeating [5,9]. For example, mindfully eating a blueberry would involve examining the berry with a beginner’s fascination, feeling its texture, smelling it, chewing it slowly, rolling the juices between one’s teeth, tuning into any sounds such as the mechanical process of chewing, and slowly swallowing it as it moves down the esophagus [6,9]. Mindful eating has been linked with reduced binge-eating behaviors [10].

One convenient medium for delivering mindfulness interventions including mindfulness-based eating awareness (MBEAT) or mindful eating is through mobile health (mHealth). mHealth is a resource for individuals that provides increased accessibility to health information and applications at any time and location using mobile phones, apps, text messages, and iPads or other tablets [11,12].

With the rise in popularity of mindfulness, there are numerous commercial mindfulness and mindful eating apps in the app stores. However, a recent review found that very few are actually based on mindfulness, and their effectiveness is unknown [13].

### Objectives

To date, there has been no review of mindful eating apps that aim to teach mindful eating with mindfulness-based eating awareness techniques, in particular, for the management of binge eating and weight management. This review aimed to better understand what types of mindful eating apps are available in the app store and to appraise their overall effectiveness. Appraising the quality of these apps includes assessing the extent to which they teach the accepted principles of mindfulness. This will provide a better understanding of whether app users who suffer from eating problems are accessing a legitimate mindful eating resource. It is also helpful for future app development and app interventions as there has not been an mHealth mindful eating app trial to date [14].

This review aimed to appraise the quality of the mindful eating apps and to appraise the quality of content on mindful eating apps.

## Methods

### Search Strategy

A review of mindful eating apps for iPhone was undertaken from March to April 2018. We searched iTunes Australia for relevant mindful eating apps using the search words, *mindful eating*.

As iTunes does not allow for string searches that may generate precise narrow search results such as in scholarly databases for literature reviews, we tested the search of *mindful eating* by first reviewing whether relevant hits came up with these terms. Once it was confirmed that these terms generated mindful eating apps and met the inclusion criteria and that other search terms did not make the search more precise or manageable, such as general *diet*, *eating*, or *mindfulness*, the final search was based on these terms. The search was carried out in English. However, mindful eating apps that were generated from the search were not excluded if they were in a different language because of the language fluency of the reviewers.

The search was limited to iTunes and thus limited to iOS to ensure budgetary compliance and manageability as we budgeted to include apps that were free up to the price of Aus $5. However, many apps in the iTunes store overlap with apps in Google Play; hence, this review has general applicability for Android users as well beside iPhone and iPad users.

### Inclusion and Exclusion Criteria

The inclusion criteria were all mindful eating apps that aimed to teach mindful eating and assist with binge eating/eating disorders with a central mindful eating–based approach or were weight loss apps whose central component focused on mindful eating. Mindful eating apps were broadly defined as apps that had any one of the following elements that are essential to mindful eating: slowing one’s eating, recording one’s time eating, being more cognizant of one’s meal and setting, using one’s 5 senses when eating, being aware of one’s intrinsic bodily hunger versus hunger because of external factors, being aware of satiety, being aware of a balanced meal, using mindful eating for weight control, and control of binge and emotional eating [2,4-9]. Our criteria were broad to ensure that we included any eating app that had a mindful eating central component or purpose.

The exclusion criteria were apps for general weight loss and eating/diet apps that did not have a central mindful eating component. Mindfulness apps that taught mindfulness meditation such as mindfulness-based stress reduction (MBSR techniques) without a central focus on mindful eating for binge/emotional eating, teaching slow eating, or weight were excluded. In addition, if apps had extra add-on features as an extra cost beyond their main standard functions, these extra add-on costs were not included for budgetary reasons.

### Appraisal

The apps were appraised for overall quality using the criteria adapted from the Mobile App Rating Scale (MARS) [15] that assesses quality on 5 domains, including engagement, information, functionality, aesthetics, and subjective impression. We also included the domains that assess if the app has a privacy policy [16] from the Enlight criteria and its therapeutic effectiveness partially adapted from the Royal College of Physicians Health Informatics unit guidelines [17]. We assessed whether the apps had included any of this or not.

Individual scores for the 5 domains in MARS were calculated by averaging the score in the questionnaire sections for each domain, which was on a scale from 1 to 5. Subjective scores were not included in the overall MARS app quality score. In addition to assessing these 4 domains for an overall MARS score, the domains of privacy and therapeutic effectiveness [16,17] as well as a consideration of user information were included in our global overall assessment score of the 5 domains.

Across the individual overall MARS and the overall global assessment with MARS scoring, we used the same cutoffs to grade the apps. Scores below 60% were weak (eg, 2/5). It should be noted that the scores were not strict whole numerical values at times as a mean score on the Likert scale was calculated from 1 to 5. For example, a mean score of 2.5/5 would also result in a score in the low percentile range less than 60%. Scores of 60% and above were considered to be moderate (eg, 3/5) and scores of 80% (eg, 4/5 in MARS or 3.5/4 in the global assessment) or above were strong. Scores above 95% (eg, 4.75/5 in MARS or 3.8/4 in the global assessment) would be classified as very strong. These cutoffs were agreed upon by the reviewers on the general basis that scores above 80% are generally considered to be good, whereas 60% represented a pass, and anything less than this was deemed to have not met at least 50% of the required criteria.

In addition to using MARS, we also appraised the apps for mindful eating against a set of domains that we agreed upon as being important to consider based on implementation by mindfulness practitioners in formal mindfulness interventions and based on research evidence drawn from the literature [2,4-9,18-21]. Mindfulness practitioners have previously recommended that individuals who struggle with eating behaviors, such as binge eating, use guided and specialized eating audios [7]. General guided audios for mindful eating have also been a part of MBSR programs, whereby the practitioner uses a fruit as an example and guides the participant through an entire mindful eating example using the 5 senses [4,6,7,9]. Our previous exploratory focus group study also found that the participants desired to have examples on how to actually practice mindfulness and eat mindfully [20]. For these reasons, we assessed whether the mindful eating apps offered any kind of guided mindful eating example. Furthermore, mindful eating involves awareness of one’s internal hunger and satiety, coupled with an awareness of external triggers, such as stress, that may influence eating [2,5,9]. Thus, the mindful eating process often involves a self-assessment of one’s hunger and whether it is because of true hunger [2,5,9]. For this reason, we appraised whether the apps helped with increasing self-awareness through an assessment of hunger (internal vs external).

Although the MARS appraisal recently added a supplementary document about assessing whether the app would change behaviors in general, as an extra add on to the scoring, we did not have access to this at the time in the original MARS scoring sheet, and it was not a part of the scoring itself [15]. Instead, we undertook a more thorough assessment of whether the apps integrated behavior change techniques (BCTs). Abraham and Michie developed a list of BCTs that are common in behavior change interventions [18]. This list includes 26 BCTs. This is essentially a qualitative appraisal that involves simply checking off a yes for any BCTs that are used in interventions [18]. There are a total of 9 domains. Weak was classified as having a total score of less than 4/9 domains (<50%). Moderate was classified as having a yes for 5 to 6 out of 9 domains. Strong was classified as having a yes for 7 to 9 of 9 domains (77%-100%).

A previous systematic review and meta-analysis of mHealth interventions for weight loss involving diet and exercise found that BCTs such as reminders, goal setting, feedback, prompts, encouragement, motivation, education, and practical tips, to name a few off the list, were commonly implemented [19]. Our previous systematic review on user perspectives on mHealth also found that users preferred BCTs in mHealth interventions [21]. Similarly, our mindfulness exploratory mHealth study also found that participants desired practical tips, reminders, encouragement, education, and motivation [20]. For these reasons, we examined whether the mindful eating apps had integrated any BCTs such as the ones described above and from the list developed by Abraham and Michie [18]. Finally, we also assessed whether mindful eating was taught through a variety of media such as audio, articles, and videos, as research indicates that individuals have different learning preferences; hence, diversity in educational materials was regarded as being desirable [22]. We also assessed the apps for quality of information in terms of whether the apps mentioned the benefits of practicing mindful eating and if they considered educating participants about a balanced diet. Our previous focus group study found that participants wished to receive information on the benefits of mindfulness and general tips about being mindful of a balanced diet [20]. In summary, we assessed whether they offered guided teaching examples, quality information about mindful eating, whether they integrated specialized eating audios, heightened self-awareness (internal hunger vs external), used a variety of media to teach mindful eating, and whether they integrated BCTs adapted from Abraham and Michie’s BCT list [18].

Two authors, LNL and SE, appraised and scored the apps. An average score was created. When there was a large disparity in the reviewer’s scoring, LNL and SE discussed the apps until there was agreement.

## Results

### Search Process

**Figure 1:**
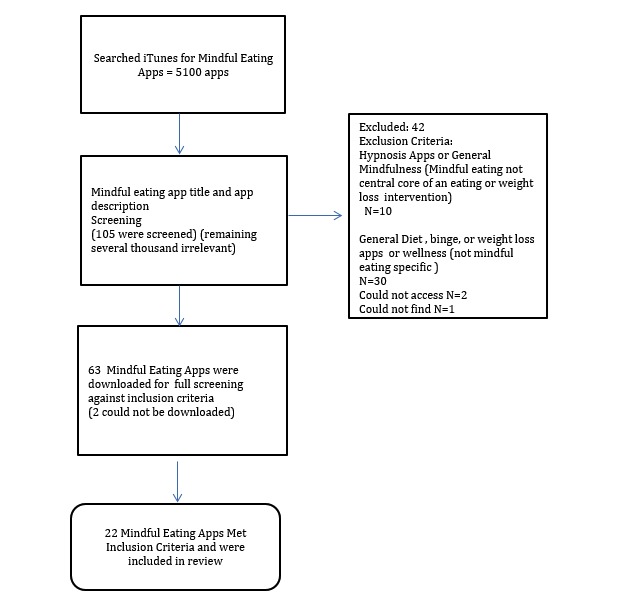
Flow chart of search process for relevant apps.

The search generated 5100 apps. It should be noted that because manual counting of the apps had to be undertaken, only an estimate was generated. However, 2 reviewers estimated the number of hits the search gave and an average was generated to represent the best approximation. After screening the app titles and app descriptions for relevancy, 63 apps (2 could not be accessed and 1 could not be found) were downloaded for further screening against the inclusion and exclusion criteria. Among them, 40 were not specific to mindful eating for binge eating, weight, or general mindful eating after reviewing their content and were excluded. A total of 22 apps met the inclusion criteria and were included in this review [23-44]. The flow chart in Figure 1 illustrates the search process.

### The Types of Mindful Eating Apps

Most of the apps (n=11) were mindful eating diaries that involved journal entries, hunger rating scales, or hunger/fullness self-assessment questions, with a few using both [1,25,38,23-42]. Some of the mindful eating apps (N=4) were mindful eating timers that measured how slowly individuals ate or focused on slow eating [28,45]. Furthermore, 3 apps were entirely audio based [27,31,41], 2 were mindful eating food menus without any mindful eating information [37,39], and 1 was based on mindful eating inspirational quotes in cards [26]. The mindful eating app descriptions are summarized in Multimedia Appendix 1.

### General App Feature Appraisal Scoring Using the Mobile App Rating Scale

The mindful eating app scores using MARS and other guidelines discussed [14,16-17] are summarized in Table 1. The mean MARS scores ranged from 1.96 (59) to 3.75 (47) out of a maximum score of 5 in the global summary score when privacy, therapeutic effectiveness, and user information was added as a domain [16-17] and out of a score of 4 in the general MARS scoring [15]. The MARS scoring system was on a 5-point scale for individual domains. The top apps receiving the highest score in the mid-3-point range out of the 5-point scale were In the Moment, Am I hungry, Weightless, and Eat-C [1,36,38,44]. None of the apps received a high-quality score of 4, overall. In addition, 3 apps received a high score, though not a very strong score on the MARS overall score, but when the domains of privacy, therapeutic effectiveness, and user information were added, the scores became moderate on the global overall score [36,38,44].

**Table 1:** General app scoring domains adapted from the Mobile App Rating Scale.

Mindful eating app	Engagement	Functionality	Information	Aesthetics	Subjective	MARSs^a^; Global score^b^	Presence of privacy policy^c^; Global score	Source of privacy policy^d^
EAT-C	2.55	4.125	2.75	3.33	2.66	3.18/4; M^e^	No; 3.18/5; M	N/A^f^
Mindfulness meals	1.55	2.52	1.65	3	1.625	2.18; W^g^	N/A; 2.18/5; W	N/A
Mindful Eating Coach: Am I Hungry?	3.55	3.75	3.75	3.4	3.515	3.6; S^h^	N/A; M	N/A
Mindful eating Tracker (Green apple icon)	3.165	3.5	2.33	3.58	2.5	3.14; M	N/A; M	N/A
The Savour Coach	3.25	3.5	2.8	2.75	2.5	3.075; M	N/A; M	N/A
Crave Mate	2.65	4	2.25	2.58	1.625	2.87; M	N/A; W	N/A
Weightless	3	4	3.18	3.35	2.125	3.38; S	N/A; M	N/A
10S Fork (related app would not load)	2.65	2.22	2.8	2.75	2.55	2.605; M	N/A; W	N/A
Intuitive	1.8	3.26	2	2.3	1.25	2.3; W	N/A; W	N/A
Lose Weight Audio Guide	2.1	2.75	2.65	3	2.625	2. 625; M	N/A; W	N/A
Mindful by Sodexo	1.65	2.375	1.35	2.5	1.125	1.96; W	N/A; W	N/A
Eat Slowly	2.35	3.75	2.4	2.165	1.75	2.66; M	N/A; W	N/A
Mindful Eating Calendar	3	3.85	2	3	2.625	2.96; M	N/A; W	N/A
Eat Breathe Thrive	2.8	1.9	2.25	2.315	2.075	2.316; W	N/A; W	N/A
In the Moment	3.55	4.25	2.8	4.36	3.275	3.74; S	N/A; M	N/A
Mindful Bite	1.9	3	2.25	3.25	2.5	2.6; M	N/A; W	N/A
Eating (Egg)	2.45	3.75	2.25	3.265	3.08	2.92; M	N/A; W	N/A
Slow Eating	2.1	3.25	2	2.53	1.35	2.47; W	N/A; W	N/A
Rise Up	3.45	3.5	2	3	1.875	2.98; M	N/A; W	N/A
Eating Thin	2.55	2.625	2.35	2.85	2.415	2.59; M	N/A; W	N/A
Jourvie	2.35	2.9	1.77	2.58	1.625	2.4; M	Yes^i^; W	Yes
Empowerment cards	2.5	4.25	2	3	2.275	2.93; M	N/A; W	N/A

^a^Mobile App Rating Scale score (mean score excluding subjective).

^b^Overall global score with privacy.

^c^Privacy policy, terms and conditions, user information, and therapeutic effectiveness [16-17]. Overall global score with these factors.

^d^Adapted from the BCTs list [18].

^e^Moderate.

^f^Not available / no.

^g^Weak.

^h^Strong.

^i^General privacy policy=1.

MARS Domain Score for Engagement, Functionality, Information, Aesthetics, and Subjective Individually Appraised: mean score of each domain criteria 5: Very strong, (VS) ≥95%; Strong (S), ≥80%; Moderate (M), 60%; and Weak (W), <60%.

MARS mean sum score/4 domains (excluding subjective): Very strong, ≥95%; Strong, ≥80%; Moderate, 60%; and Weak <60%.

Overall score with privacy policy added as an extra domain/5 domains: Very strong ≥95%; Strong, ≥80%; Moderate, 60%; and Weak <60%.

The majority of the apps functioned well; hence, they received good scores for functionality. Specifically, a total of 5 received strong scores (22%) [26,29,35,38,44] and 9 received moderate scores in the higher end (>3/5) nearing strong (41%) [23,25,27,32-34,36,43], with the rest receiving moderate scores in the lower range of the 3-point scale of 5 or low scores in the higher range of 2/5. However, a few (18%) received low scores in the lowest point score range of less than 2/5 as they were slow or some of the content did not load or moving between sections was difficult when returning to the home screen (back button had to be used if there was no home screen icon) [28,39,42,45]. In terms of aesthetics, most of the apps were average, mostly receiving scores of above 3 in this domain (n=11) [23-26,29,31,32,36,37,43,44], whereas others received weaker scores in the range of 2/5. One app received a high score in this domain [38]. They were neither visually unpleasant nor extraordinary in their presentation with nice designs or specialized graphics or animations. They generally used a standard color with a basic design. Most of the apps received weak scores for information in the point range of 2/5 (19/22, 86%) [23-28,31,33,35,37,39-43,45]. Very few had any informational content in them and were mostly tracking apps for slow eating or journal entry apps. Thus, the quality of the information and the quantity of the information were weak overall. A few received higher scores for showing graphs based on users’ entries for their mindful eating.

The apps also received lower scores for engagement as few were entertaining for longer than 5 min and were mostly uninteresting. A total of 14/22 apps (63%) [23,25,26,28,29,31,34,35,37,39-42,45] received low scores for engagement, with the rest receiving moderate scores. In terms of customization, a few apps allowed users to select how often they wanted notifications, but they lacked in providing personalized messages specific to mindful eating.

In terms of credibility and evidence, these apps were not tested in trials. In addition, most of the apps did not have an information section icon with instructions or any kind of privacy policy section. Little is known about their therapeutic effectiveness as they have not been trialed.

### Mindful Eating–Specific Content Assessment of the Apps

Overall, the majority of mindful eating apps (19/22, 86%) received weak scores in the mindful eating–specific assessment content of the apps of less than 4/9 domains (<50%) summarized in Multimedia Appendix 2 [23-29,31-35,37,39-43,45].

#### Assessment of Diversity of Media to Teach Mindfulness

Assessment of the mindful eating content of the apps is summarized in Multimedia Appendix 1. There were very few apps that contained multiple features for mindful eating. Most of them only used 1 type of medium, which was mostly written content or space for writing by the users. Out of the apps, only 3 (3/22, 1%) had any meditation audios, and these apps lacked other content such as written content or videos [27,31,41].

#### Assessment of Quality of Information

##### Mindful Eating Examples

Most apps had little information about mindful eating, including guided examples using a food meditation example by tuning into all 5 senses when eating. Only 2 considered the senses but not in detail [27,43]. None had specially tailored mindful eating audios for hunger, fullness, and binge eating, apart from the few apps that had any audio. However, 1 app mentioned assessing hunger in different body parts such as the eyes and heart [25] and another had a brief note about using the senses without a thorough guided example [43].

##### Information on the Benefits of Mindful Eating and a Balanced Diet

The apps did not mention the benefits of mindful eating on health in anything more than a vague form. The main emphasis was mostly on counting how slowly one ate. Most of them lacked general information about a healthy balanced diet, including the World Health Organization’s target guideline information [46,47]. Specifically, only 4 apps (4/22, 18%) considered teaching users about some form of healthy eating directly in the app itself [23,35,38,44]. However, this information was very general. In addition, 1 app had links to information on a website [36].

#### Assessment of Internal Versus External Hunger Cues

Overall, 10 apps allowed (45%) users to reflect on their eating motives such as stress versus internal hunger by ratings [42,43] or by audio [27], general hunger motives [1,25,36], and a few asked about general emotions and cravings [23,35,44,45].

#### Assessment of Integration of Behavior Change Techniques

Furthermore, 6 apps (27%) had integrated a range of BCTs [29,38,36,25,43,44]. The most common BCTs in the reviewed apps involved self-monitoring, such as self-monitoring of mindful eating through a journal, hunger rating, or using an eating timer. None of the apps had push notifications that had specialized or tailored messages for mindful eating specifically rather than general reminders. The apps did not offer tips or advice for integrating mindful eating into users’ daily lives.

## Discussion

### Summary and Implications of Findings

In summary, this was the first review to appraise mindful eating apps for their overall quality. We aimed to appraise the quality of the mindful eating apps for iOS in the iTunes store by using the MARS appraisal tool [15]. We further appraised the quality of content on mindful eating apps using a set of criteria agreed upon by the reviewers as being important based on the relevant literature and based on mindful eating practices taught by practitioners [4-10,18-20,48].

Overall, most of the apps received a weak global summary score when considering the key domains in MARS [15]. This included a consideration of the domains of privacy, information policy, and therapeutic effectiveness. Most of the apps had an overall weak-to-moderate MARS score as well. The weakest MARS areas were in the quality of information and engagement. Most of the apps were average in terms of aesthetics. Most apps received strong scores for overall functionality as there were no major technical functionality issues that we had encountered. In addition to this, our assessment of the content of mindful eating apps found that most of the apps received weak scores, overall, for the quality of content on mindful eating. The areas that needed improvement included providing more information about mindful eating such as using mindful eating examples that involved tuning in with all 5 senses, teaching users to assess their internal hunger and satiety cues, offering real-life mindful eating practical tips and advice, using a variety of media to teach mindfulness, and integrating a range of BCTs. The implications of these findings are discussed below.

Although there are many apps that claim to be based on mindful eating, few comprehensively cover the essential aspects of mindful eating or MBEAT. The review by Mani et al also found that less than 5% of mindfulness app are truly based on mindfulness [13]. Mindful eating requires attuning oneself to all 5 senses when eating by really focusing on the sight, smells, sounds, flavors, and physical sensations of the food one is eating, without allowing one’s thoughts to wander [6,9]. It is essentially a type of eating meditation [4,7].

Most of the apps tracked how slowly someone ate. Eating slowly is indeed an important aspect of mindful eating [4-9]. Although mindful eating involves slow eating, there is much more to a mindful bite than simply chewing slowly because if an individual is not truly present and all of their 5 senses are not immersed in the experience, they are just eating slowly but not mindfully [4-9]. Thus, mindful eating apps need to do more than track slow eating or hunger and fullness ratings. They need to ask users about what they are tasting, sensing, feeling, seeing, and hearing when eating as these are essential to the mindful eating process according to mindful eating practitioners and the literature [4-9]. This could be in the form of ratings or through journal entries that allow users to reflect on their mindful eating after they listen to a mindful eating meditation audio, for instance.

Furthermore, none of the apps guided users through a mindful eating example, with the exception of 1 audio app that was only audio based. Guiding users through an eating meditation is important to teach them how to truly eat mindfully as in the leading books made by mindfulness practitioners that have accompanying audios [4,6]. For example, in Dr Kabat-Zinn’s introductory book, he guides users through the whole sensory mindful eating meditation experience, which may be listened to with the accompanying CD [4]. Mindfulness practitioners also provide tailored binge eating, hunger, and fullness meditation audios that are valuable, yet none of the reviewed apps had these options [7]. This would be valuable for the apps that claim to assist with binge eating in particular.

In addition, having a journal alongside an eating audio would help users to record their experiences during a mindful eating meditation as this is found in traditional mindfulness-based training book programs [6]. If the apps do not thoroughly explain what mindful eating is and guide users through an example, the potential for knowledge acquisition and proper mindful eating practice may be limited.

Mindful eating can be as detailed as paying attention to the entire meal setting itself including the dining room, the placement of cutlery, and the movement of one’s hands [8]. Mindful eating can also involve trying different foods and discerning between the different textures and flavors with closed eyes to increase one’s taste bud sensitivity, which enhances the mindful eating experience, as a leading mindful eating practitioner recommends [8]. Integrating these types of examples in an app would be insightful. Only 1 app mentioned paying attention to cutlery and the meal setting very briefly [48]. Thus, mindful eating apps could integrate these components and tips in their apps to further stimulate mindful eating and interest. In addition, practical real-life suggestions could help users with eating mindfully throughout the day at work, out with friends socially, and at home. Practical real-life suggestions were found to be important for participants in our previous exploratory pilot study [20].

A variety of media to teach mindful eating would be desirable as individuals have different learning preferences, which can include learning through listening or watching according to research on learning styles [22]. Furthermore, research suggests that most individuals stop using their apps because of boredom, costs, and having to manually key in data [49]. As most apps had weak scores in the engagement and information domains in the MARS appraisal, using diverse media could enhance the informational content and provide more user engagement. Most of the apps were uninteresting, and it is unlikely that users would continue using them for longer periods of time. Hence, ensuring the app is entertaining and fun and has a variety of content is key to sustainability. Only 1 app had a reward [38]. Most were either boring diaries that required users to manually key in data or timers that did not have any other information or content. The review of mindfulness apps similarly found that most apps had weak scores in the engagement and information domains [13]. Our exploratory pilot qualitative study also found that users expressed a preference for mindfulness apps that could provide real-life exercises that are entertaining and relevant, with plenty of practical educational content [20]. Thus, it seems that developers could work on ensuring higher quality of information and user engagement.

In addition, few apps integrated a range of BCTs. Our postrandomized controlled trial focus group results indicate that learning mindfulness requires habit formation, which in turn not only takes time but also requires reminders [48]. Our pretrial exploratory study also found that students expressed a preference for a range of BCTs that also included prompts, education, tips, reminders, encouragement, and motivation, to name a few [20]. Moreover, our systematic review of consumer preferences further found that BCTs are desired by participants in mHealth weight loss interventions [21], and they have been commonly integrated in past mHealth interventions for weight loss involving diet and exercise [19]. Thus, future mindful eating apps could consider integrating a range of BCTs.

The push notifications also were not mindful eating specific nor were there any practical tips on how to eat mindfully throughout the day. To truly motivate individuals and remind them to eat mindfully, messages should aim to be mindful eating specific, ideally with practical tips [20]. We found that participants in our previous study expressed a preference for mindfulness-based messages with practical tips on how to practice mindfulness in their daily lives [20]. Thus, future apps could consider constructing mindfulness-based messages that would have real-life mindful eating practical tips in them.

In addition, there is a need to further improve the apps in the information domain. Most of the apps also did not fully explain the benefits of mindful eating for users such as reduced binge, emotional eating, stress, and weight [3,50,51]. It is important for users to know why they should aim to eat mindfully because being aware of the benefits could motivate them to practice it. Our previous focus group exploratory pilot study found that participants needed more information about the benefits of mindfulness practice to be mindful [20]. Thus, the apps could provide some basic information from the literature that has linked mindful eating with improved binge eating [50] and reduced stress [3], for example. This would make the app more relevant for individuals with specific problems that the app could address.

Furthermore, although mindful eating does not require caloric restriction [5], to achieve a balanced diet and make mindful decisions about one’s nutrition, there should be basic information in an app. According to Kristeller and Ruth, mindful eating requires a general knowledge of what is healthy food and what one should aim to meet [52]. We also found in our focus group study that students wanted more general health and nutritional information [20]. Thus, future apps could consider providing basic information about a balanced diet.

Mindful eating apps should also provide more information on stress eating and how to assess if one is eating in response to external stressors or internal hunger as not all apps considered this. Why is this important? Mindful eating practitioners often ask participants to rate their hunger and appraise whether it is because of true internal hunger and satiety or because of extrinsic reasons as this increases present moment self-awareness [2,5,6,8,9]. Increased present-moment awareness may in turn lead to improved eating behaviors such as reduced binge eating [2,50]. Research on stress indicates that stress is one of the determinants of unhealthy eating behavior [53-56] and that mindfulness practice assists with stress and eating behaviors [3,50]. Special breathing techniques could be taught in the app that help users breathe out the craving and relax, hence, minimizing binge or emotional eating as leading mindfulness practitioners recommend [7]. Although some apps provided visual feedback for when users said they ate slowly, receiving feedback on actual emotional states and stress levels before and after meals in tandem with logging breathing exercises for craving control could be helpful, for instance, as emotional awareness and awareness of internal bodily cues during eating are part of the mindful eating process [2,5].

Finally, most of the apps did not have information on privacy policies, which is an important requisite [16]. A review found that 81% of diabetes apps did not have privacy policies and that 76% of diabetes apps that did not have privacy policies had distributed sensitive information to others [57]. A recent review of mental health apps for bipolar disorder also found that most of the apps did not have privacy policies nor had they cited their informational resources [58]. This raises serious ethical and safety concerns surrounding privacy and confidentiality as individuals do not have the opportunity to provide their informed consent which is the basis for health ethical practice [59] and should be applicable to health promotion and mental health apps as they deal with health information, yet reviews have found that these apps have not provided privacy policies [57,58]. This is especially concerning when sensitive health data are potentially shared with third parties, and research indicates that 7 out of 10 apps shared data with third parties [60]. Although apps collect things such as location or have access to one’s camera [61], they may have access to more sensitive health information if this information is stored and recorded by the individual. Although privacy is a human right [62], research also indicates that privacy laws in countries with strict regulation do not protect citizens when their information is passed on to third parties in other countries [60]. Thus, future mindful eating apps should ensure that they have clear privacy policies. iTunes now has a requirement for all apps to have a privacy policy to be accepted [63].

In addition to the lack of privacy policies, the apps also lacked information on proven therapeutic effectiveness. Hence, future researchers should undertake research to determine what the evidence base really is for commercial mindful eating apps. In other words, are they effective?

### Strengths and Limitations

Although this is the first review to appraise mindful eating apps for iOS, Android apps were not reviewed. As a result, there is always a possibility that we may have missed additional apps. However, we believe we have included the main mindful eating apps available in the marketplace. There is also an overlap in apps between iTunes and Google Play. Furthermore, it is possible that the expensive mindfulness subscription apps may have mindful eating components as add-on features, but our focus was on mindful eating apps made for the primary purpose of teaching mindful eating and assisting with binge eating or weight management.

### Conclusions

We undertook the first review of mindful eating applications for iOS. We found that many of the mindful eating apps did not have sufficient information on how to eat mindfully to deliver appropriate mindful eating training. Most of them lacked in comprehensiveness and were mostly eating timers, diaries, or hunger rating scales. There was very little information on how to eat mindfully or how to make mindful eating decisions when confronted with cravings. There was little diversity in app media and little that could increase engagement in the user experience. Most of them also did not offer mindful eating meditations or guided examples, and there was little information on privacy for users. Future mindful eating apps could be improved by developing these various domains.

## Appendix

Multimedia Appendix 1 Mindful eating apps description.

Multimedia Appendix 2 Mindful Eating Specific Content Assessment of Apps.

## Abbreviations

BCT: behavior change technique
MARS: Mobile App Rating Scale
mHealth: mobile health
