# Supplementary figures and images for: Mindful Eating Mobile Health Apps: Review and Appraisal
Source: JMIR Ment Health. 2019 Aug 22;6(8):e12820. doi: 10.2196/12820 (PMC6727629; doi:10.2196/12820)

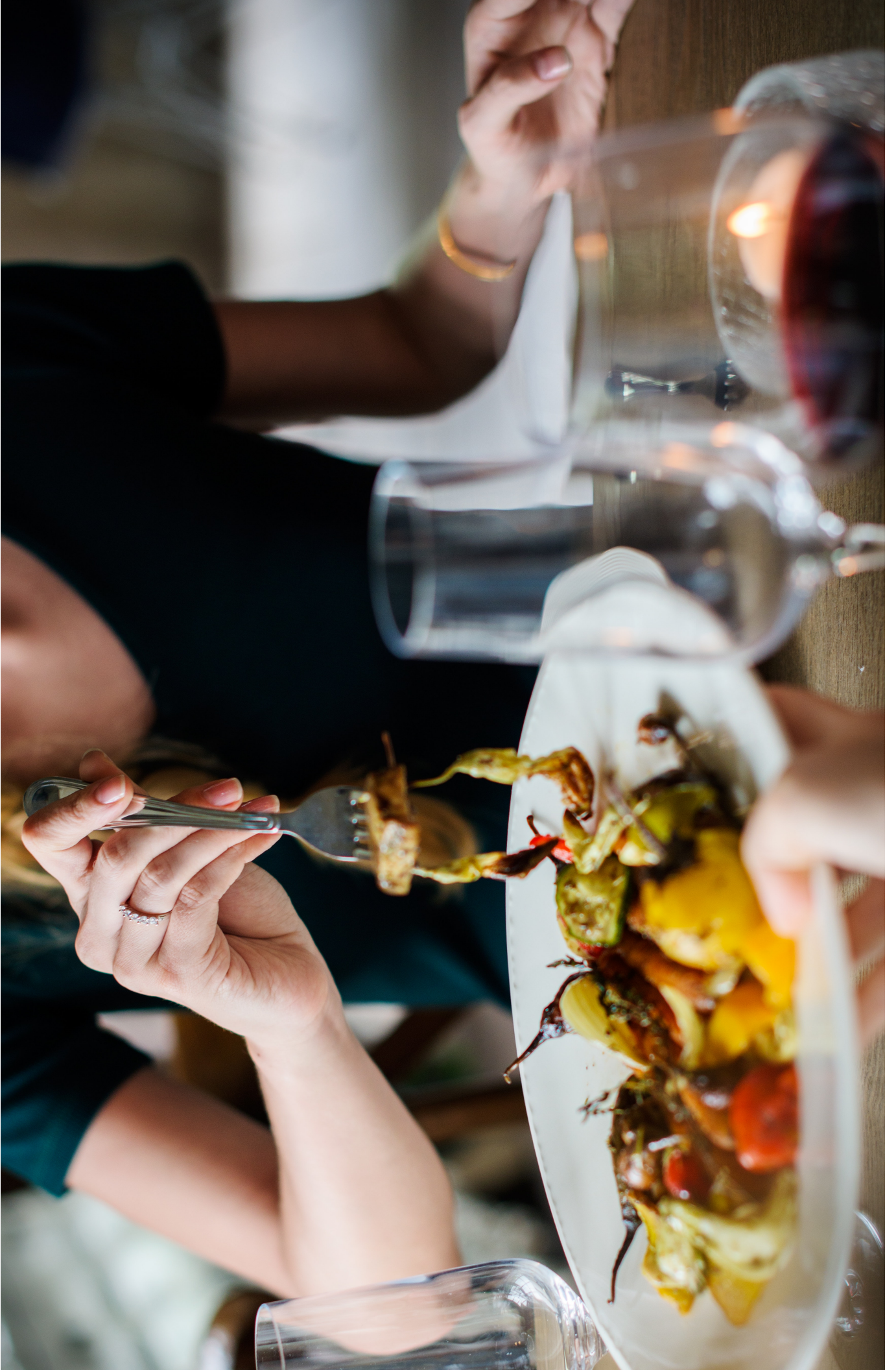

Supplement: Multimedia Appendix 1 [file mental_v6i8e12820_app1.pdf]
