# Supplementary material for: Mindful Eating Mobile Health Apps: Review and Appraisal
Source: JMIR Ment Health. 2019 Aug 22;6(8):e12820. doi: 10.2196/12820 (PMC6727629; doi:10.2196/12820)
Supplement: Multimedia Appendix 2 [file mental_v6i8e12820_app2.pdf]

Weak (W)= Yes <4/9 domains Moderate (M)= Yes 5-6/9 domains Strong(S)=Yes 7-9/9 domains

Table Mindful Eating Specific Content Assessment of Apps

Mindful Eating App	Teaches Mindful Eating with guided examples or eating meditation	Increases self-awareness +reflection on eating motives (internal vs. external)	Uses a variety of media to teach (audio, videos, articles)	Offers specialized tailored meditation for eating: binge, hunger + satiety	Offers real life advice how to eat mindfully /different settings	Provides reminders and tips for mindful eating	Allows for mindful eating self-monitoring , goal setting, /reflection with feedback?	General info on being mindful of a balanced diet /extra resources	Range of BCTS ? (18)	Quality
EAT-C	Basic info what it is and what it is not (not detailed) No guided examples of using five senses (only savour food) or mindful eating meditation	Meal Hunger Fullness Scale (no scale on internal hunger versus external stress eating)but has the Meme BEMINDFUL(explore non-hunger reasons)	No	No	No	No	Yes provides graphic feedback over weeks , goal setting	No diet/guideline info	Self-monitoring goal setting , feedback	W
Mindfulness Meals	No mindful eating info	No	No	No	Offers food recipes, no mindful eating tips	Yes but not specific for mindful eating	No	Healthy meal recipe info but no info on	No	W

Mindful Eating App	Teaches Mindful Eating with guided examples or eating meditation	Increases self-awareness +reflection on eating motives (internal vs. external)	Uses a variety of media to teach (audio, videos, articles)	Offers specialized tailored meditation for eating: binge, hunger + satiety	Offers real life advice how to eat mindfully /different settings	Provides reminders and tips for mindful eating	Allows for mindful eating self-monitoring , goal setting, /reflection with feedback?	General info on being mindful of a balanced diet /extra resources	Range of BCTS ? (18)	Quality
							Self-monitoring	general guidelines/n utrition		
Mindful Eating Tracker (green apple icon)	No guided Mindful eating info (mentions hunger awareness of the nose, heart, eyes , etc.), No food eating guided examples or eating meditation (Agree with this)	Hunger rating scale (no assessment of internal versus external drivers)<br><br>Yes, agree	No	No	No	Reminders	Calendar for Self-monitoring, feedback with graphs, reminders, personalization	No	Yes	W
The Savour	Guided audio	Self-	No	Starts with	No	Push	No	No	No	W

Mindful Eating App	Teaches Mindful Eating with guided examples or eating meditation	Increases self-awareness +reflection on eating motives (internal vs. external)	Uses a variety of media to teach (audio, videos, articles)	Offers specialized tailored meditation for eating: binge, hunger + satiety	Offers real life advice how to eat mindfully /different settings	Provides reminders and tips for mindful eating	Allows for mindful eating self-monitoring , goal setting, /reflection with feedback?	General info on being mindful of a balanced diet /extra resources	Range of BCTS ? (18)	Quality
Coach	of mindful eating (no general written info)	awareness via audio (Body scan and insight into stress eating (what is eating you)		body scan audio to relax (not really binge specific)		notifications ; no mindful eating tips				
10S Slow Fork	Discusses benefits of slow eating but not the core principles of ME	Just awareness of slow-eating	No	No	No	N/A	Self-monitoring of slow eating daily	No	No	W

Mindful Eating App	Teaches Mindful Eating with guided examples or eating meditation	Increases self-awareness +reflection on eating motives (internal vs. external)	Uses a variety of media to teach (audio, videos, articles)	Offers specialized tailored meditation for eating: binge, hunger + satiety	Offers real life advice how to eat mindfully /different settings	Provides reminders and tips for mindful eating	Allows for mindful eating self-monitoring , goal setting, /reflection with feedback?	General info on being mindful of a balanced diet /extra resources	Range of BCTS ? (18)	Quality
Intuitive	No mindful eating guided info	No, general feedback based on eating input	No	No	No	No just recommendations to eat mindfully	No real self-monitoring graphs	No	No	W
Mindful by Sodexo	No	No	No	No	No just meal tips	Push notifications but not ME specific	No	No just a food menu	No	W
Eat Slowly	No just times meals (1 paragraph on benefits of slow eating)	No but some awareness of eating speed	No	No	No	No	Allows for slow eating tracking	No	No	W
Mindful Eating Calendar	Website has basic info but not in English but app doesn't	Yes : requires a motives description (brief food	No	No	No	Has push notifications	Self-monitoring in the journal	No but has a website link with info but in German	No	W

Mindful Eating App	Teaches Mindful Eating with guided examples or eating meditation	Increases self-awareness +reflection on eating motives (internal vs. external)	Uses a variety of media to teach (audio, videos, articles)	Offers specialized tailored meditation for eating: binge, hunger + satiety	Offers real life advice how to eat mindfully /different settings	Provides reminders and tips for mindful eating	Allows for mindful eating self-monitoring , goal setting, /reflection with feedback?	General info on being mindful of a balanced diet /extra resources	Range of BCTS ? (18)	Quality
Eat Breathe Thrive	No	description social /habit compensator y reflection) Assess emotional vs. hunger yes	No- audio not working??	No	No	No	No	No	No	W
Mindful Bite	No	No	No	No	No	No	No	No	No	W
Crave Mate	No (just a note on mindful eating)	Self-reflection on where cravings came from	No	No	No	Yes can set reminders	General goals listed by app for users (not really ME specific)	General wellness goals (some diet/PA info)	No	W

Mindful Eating App	Teaches Mindful Eating with guided examples or eating meditation	Increases self-awareness +reflection on eating motives (internal vs. external)	Uses a variety of media to teach (audio, videos, articles)	Offers specialized tailored meditation for eating: binge, hunger + satiety	Offers real life advice how to eat mindfully /different settings	Provides reminders and tips for mindful eating	Allows for mindful eating self-monitoring , goal setting, /reflection with feedback?	General info on being mindful of a balanced diet /extra resources	Range of BCTS ? (18)	Quality
Eating Thin	Sweets audio meditation (more based on mindfulness than ME sweet example)	It increases general bodily self-awareness using mindfulness (but not really ME specific)	No	No(but the other audios are for extra purchase did not buy extra)	No	No	No	No	No	W
Jourvie	Not really just a hunger/fullness rating and meal log	Assesses emotions (recall feelings before bingeing)		No	No	Can set reminders	No feedback	No	No	W
Empowerment cards	Cards that offer mindful eating advice	Partly through reading the cards (no features to assess one's hunger or ME)		No	Some practical tips in the cards	Can set reminders (e.g. eat slowly)	No	No	No	W

Mindful Eating App	Teaches Mindful Eating with guided examples or eating meditation	Increases self-awareness +reflection on eating motives (internal vs. external)	Uses a variety of media to teach (audio, videos, articles)	Offers specialized tailored meditation for eating: binge, hunger + satiety	Offers real life advice how to eat mindfully /different settings	Provides reminders and tips for mindful eating	Allows for mindful eating self-monitoring , goal setting, /reflection with feedback?	General info on being mindful of a balanced diet /extra resources	Range of BCTS ? (18)	Quality
Slow Eating	No just a timer With meal entry-(how fast did you eat)	Only slow eating awareness + questions on feelings when eating	No	No	No	Reminder to eat slowly	Slow eating self-monitoring only	No	No	W
Egg	No	General appraisal of emotions	No	No	No	No	No	General diet/wellness info	No	W
Lose Weight Audios	Yes	Not really but audios increase awareness of eating	No audios only	Mindful eating varieties	No	reminders	No	No	No	W-M
Rise Up	Small ME component (brief explanation using the senses without a piece of food for	Yes questions on physical hunger versus emotional (describe emotions)	No	No	No	Yes notifications	Meal logs and hunger	N/A?	Yes Self-monitoring reminders, education	W-M

Mindful Eating App	Teaches Mindful Eating with guided examples or eating meditation	Increases self-awareness +reflection on eating motives (internal vs. external)	Uses a variety of media to teach (audio, videos, articles)	Offers specialized tailored meditation for eating: binge, hunger + satiety	Offers real life advice how to eat mindfully /different settings	Provides reminders and tips for mindful eating	Allows for mindful eating self-monitoring , goal setting, /reflection with feedback?	General info on being mindful of a balanced diet /extra resources	Range of BCTS ? (18)	Quality
	demonstration; no guided mindful eating meditation audio example									
Am I hungry	Online program but no ME examples in the actual app (ME cycle general info) Discusses meal/dinning setting (fork placement) No guided audio ME meditation or example using five senses	Uses a Hunger scale (no scale for internal versus external eating) but asks to reflect /be self-aware on what user needs (cravings versus bodily needs) (yes)	In the app No but has links to a website	No	No diet guideline target info but advises to have a balanced diet (General self-care info)	Yes prompts e.g. do a heart body scan	Yes Self-monitoring	No but online program on food	Yes	M

Mindful Eating App	Teaches Mindful Eating with guided examples or eating meditation	Increases self-awareness +reflection on eating motives (internal vs. external)	Uses a variety of media to teach (audio, videos, articles)	Offers specialized tailored meditation for eating: binge, hunger + satiety	Offers real life advice how to eat mindfully /different settings	Provides reminders and tips for mindful eating	Allows for mindful eating self-monitoring , goal setting, /reflection with feedback?	General info on being mindful of a balanced diet /extra resources	Range of BCTS ? (18)	Quality	
In the Moment	Gives examples how to approach stress eating and be mindful with breathing (no Mindful guided eating example)	Yes	Offers audios to click on (meditatio n)	No	Offers general practical tips	Yes	No journal for monitoring but allows users to select scenarios that apply and offers rewards	Very general tips	Rewards	M	
Weightless	No info on mindful eating with examples or benefits etc	Yes allows for scoring of (I agree with your statement cravings, time spent eating, journal	No	No	No	Wellness coach gives general motivation	Yes self-monitoring of eating speed, cravings, etc	General wellness coach	Yes goal setting, reminders, prompts, feedback, education	M	M

Mindful Eating App	Teaches Mindful Eating with guided examples or eating meditation	Increases self-awareness +reflection on eating motives (internal vs. external)	Uses a variety of media to teach (audio, videos, articles)	Offers specialized tailored meditation for eating: binge, hunger + satiety	Offers real life advice how to eat mindfully /different settings	Provides reminders and tips for mindful eating	Allows for mindful eating self-monitoring , goal setting, /reflection with feedback?	General info on being mindful of a balanced diet /extra resources	Range of BCTS ? (18)	Quality

*(18) adapted from BCTs list
